# Supplementary material for: Towards middle-up analysis of polyclonal antibodies: subclass-specific N-glycosylation profiling of murine immunoglobulin G (IgG) by means of HPLC-MS
Source: Sci Rep. 2020 Oct 22;10:18080. doi: 10.1038/s41598-020-75045-1 (PMC7581757; doi:10.1038/s41598-020-75045-1)
Supplement: Supplementary file 1 — Supplementary Information 1. [file 41598_2020_75045_MOESM1_ESM.pdf]

SUPPLEMENTARY INFORMATION

Towards middle-up analysis of polyclonal antibodies: subclass-specific *N*-glycosylation profiling of murine immunoglobulin G (IgG) by means of HPLC-MS

Constantin Blöchl[1], Christof Regl[1,2], Christian G. Huber[1,2], Petra Winter[3], Richard Weiss[3], and Therese Wohlschlager[1,2,*]

[1]Department of Biosciences, Bioanalytical Research Labs, University of Salzburg, Hellbrunner Straße 34, 5020 Salzburg, Austria

[2]Christian Doppler Laboratory for Innovative Tools for Biosimilar Characterization, University of Salzburg, Hellbrunner Straße 34, 5020 Salzburg, Austria

[3]Department of Biosciences, Division of Allergy and Immunology, University of Salzburg, Hellbrunner Straße 34, 5020 Salzburg, Austria

*Correspondence should be addressed to therese.wohlschlager@sbg.ac.at

Table of contents

Figure S-1: Schematic workflow for SpeB cleavage site determination in HC portions Page S-2

Figure S-2: Amino acid sequences of HC constant regions of murine IgG subclasses Page S-3

Figure S-3: Fragment ion spectrum of tryptic peptide [269-286] of IgG1 from BALB/c Page S-4

Figure S-4: Separation and quantification of main and minor Fc/2 cleavage products Page S-5

Figure S-5: Fractional subclass abundances of total IgGs from BALB/c and C57BL/6 Page S-6

Figure S-6: Raw mass spectra of IgG1, IgG2a and IgG2b from BALB/c Page S-7

Figure S-7: Identification of oxidised tryptic peptide [179-194] of IgG1 from BALB/c Page S-8

Figure S-8: Deconvoluted mass spectra of Fc/2 from monoclonal IgG1 and IgG2b Page S-9

Figure S-9: Schematic workflow for Phl p 6 vaccination study Page S-10

Figure S-10: IgG subclass abundances after Phl p 6 vaccination Page S-11

Figure S-11: Subclass-specific glycosylation profiles of total IgG after Phl p 6 vaccination Page S-12

Figure S-12: Glycoform abundances of IgG1 from mouse individuals Page S-13

Table S-1: SpeB cleavage products of murine IgG subclasses Page S-14

Table S-2: *N*-glycan structures considered in glycoform assignment Page S-15

SI References Page S-18

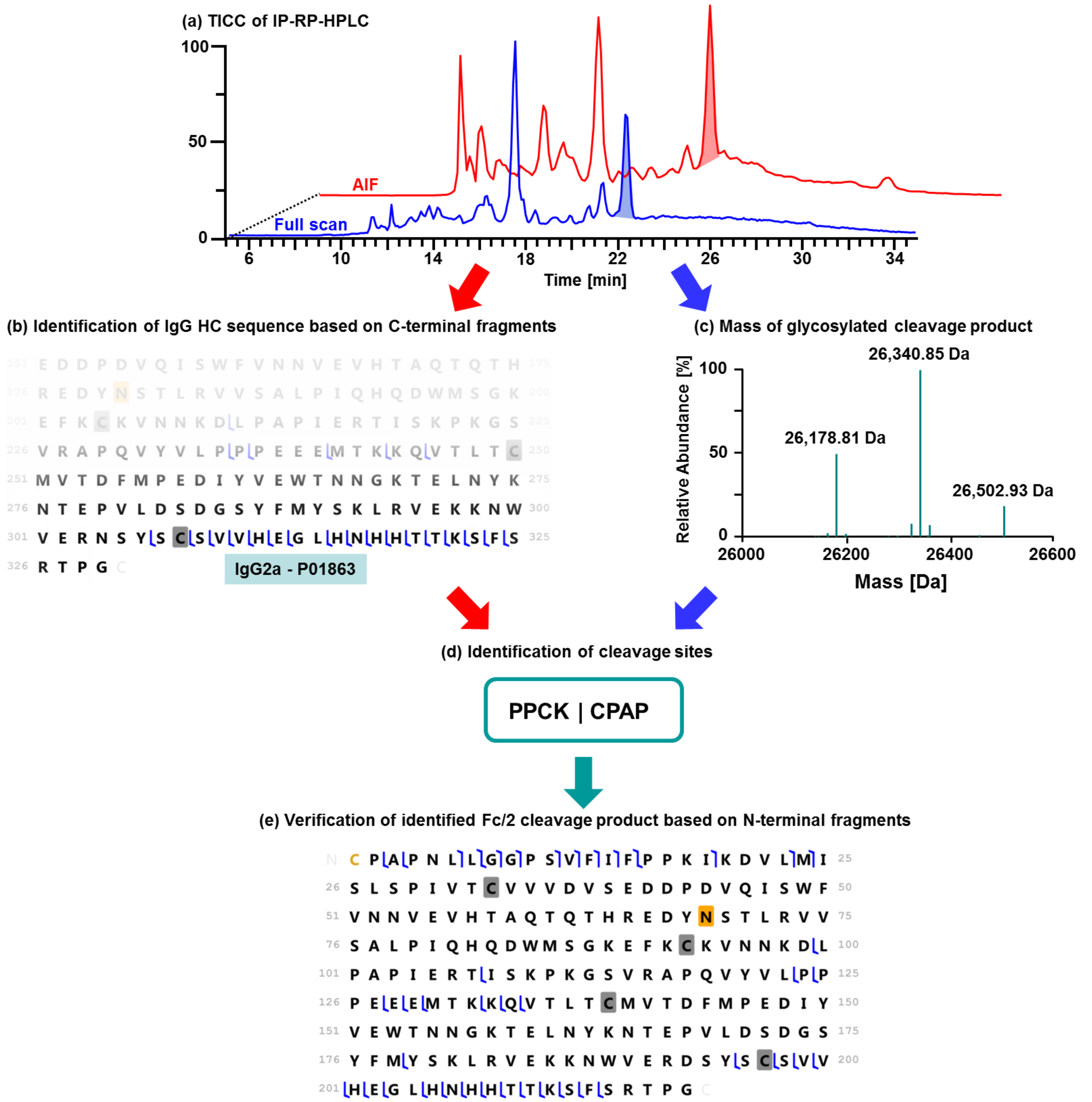

Figure S-1. Schematic workflow for SpeB cleavage site determination in HC portions of IgG as shown for polyclonal IgG2a (P01863) from BALB/c. (a) SpeB cleavage products are separated by IP-RP-HPLC and detected in full MS/AIF mode. The AIF trace is depicted at a time offset of 4.0 min. (b) Fragment spectra obtained by AIF are deconvoluted and searched against the sequences provided in Fig. S-2 using the ProSight software.[1] The constant HC sequence is identified based on the assigned y-ions, i.e. C-terminal fragments (blue marks). (c) Masses of the glycosylated cleavage product are derived from the corresponding full scan spectra. (d) The cleavage site is determined based on the mass of the obtained proteolytic product taking into account the G1F glycoform. The process is supported by the GPMAW software.[2] (e) For further confirmation, the sequence of the identified proteolytic product is searched against AIF spectra to match b-ions, i.e. N-terminal fragments, using the ProSight software. Fragmentation data for all identified cleavage sites are available as Supplementary Excel file.

IgG1 (P01868)

```
1       AKTTPPSVYP LAPGSAAQTN SMVTLGCLVK GYFPEPVTVT WNSGSLSSGV HTFPAVLQSD
61      LYTLSSSVTV PSSPRPSETV TCNVAHPASS TKVDKKIVPR DCGCKPCICT VPEVSSVFIF
121     PPKPKDVLTI TLTPKVTCVV VDISKDDPEV QFSWFVDDVE VHTAQTQPRE EQFNSTFRSV
181     SELPIMHQDW LNGKEFKCRV NSAAFPAPIE KTISKTKGRP KAPQVYTIPP PKEQMAKDKV
241     SLTCMITDFF PEDITVEWQW NGQPAENYKN TQPIMDTDGS YFVYSKLNVQ KSNWEAGNTF
301     TCSVLHEGLH NHHTEKSLSH SPGK
```

IgG1i (A0A075B5P4)

```
1       XKTTPPSVYP LAPGSAAQTN SMVTLGCLVK GYFPEPVTVT WNSGSLSSGV HTFPAVLQSD
61      LYTLSSSVTV PSSTWPSQTV TCNVAHPASS TKVDKKIVPR DCGCKPCICT VPEVSSVFIF
121     PPKPKDVLTI TLTPKVTCVV VDISKDDPEV QFSWFVDDVE VHTAQTKPRE EQINSTFRSV
181     SELPIMHQDW LNGKEFKCRV NSAAFPAPIE KTISKTKGRP KAPQVYTIPP PKEQMAKDKV
241     SLTCMITNFF PEDITVEWQW NGQPAENYKN TQPIMDTDGS YFVYSKLNVQ KSNWEAGNTF
301     TCSVLHEGLH NHHTEKSLSH SPGK
```

IgG2a (P01863)

```
1       AKTTAPSVYP LAPVCGDTTG SSVTLGCLVK GYFPEPVTLT WNSGSLSSGV HTFPAVLQSD
61      LYTLSSSVTV TSSTWPSQSI TCNVAHPASS TKVDKKIEPR GPTIKPCPPC KCPAPNLLGG
121     PSVFIFPPKI KDVLMISLSP IVTCVVVDVS EDDPDVQISW FVNNVEVHTA QTQTHREDYN
181     STLRVVSALP IQHQDWMSGK EFKCKVNNKD LPAPIERTIS KPKGSVRAPQ VYVLPPPEEE
241     MTKKQVTLTC MVTDFMPEDI YVEWTNNGKT ELNYKNTEPV LDSDGSYFMY SKLRVEKKNW
301     VERNSYSCSV VHEGLHNHHT TKSFSRTPGK
```

IgG2b (P01867)

```
1       KTTPPSVYPL APGCGDTTGS SVTLGCLVKG YFPESVTVTW NSGSLSSSVH TFPALLQSGL
61      YTMSSSVTVP SSTWPSQTVT CSVAHPASST TVDKKLEPSG PISTINPCPP CKECHKCPAP
121     NLEGGPSVFI FPPNIKDVLM ISLTPKVTCV VVDVSEDDPD VQISWFVNNV EVHTAQTQTH
181     REDYNSTIRV VSTLPIQHQD WMSGKEFKCK VNNKDLPSPI ERTISKIKGL VRAPQVYILP
241     PPAEQLSRKD VSLTCLVVGF NPGDISVEWT SNGHTEENYK DTAPVLDSDG SYFIYSKLNM
301     KTSKWEKTDS FSCNVRHEGL KNYYLKKTIS RSPGK
```

IgG2bi (A0A075B5P3)

```
1       XKTTPPSVYP LAPGCGDTTG SSVTLGCLVK GYFPESVTVT WNSGSLSSSV HTFPALLQSG
61      LYTMSSSVTV PSSTWPSQTV TCSVAHPASS TTVDKKLEPS GPISTINPCP PCKECHKCPA
121     PNLEGGPSVF IFPPNIKDVL MISLTPKVTC VVVDVSEDDP DVRISWFVNN VEVHTAQTQT
181     HREDYNSTIR VVSALPIQHQ DWMSGKEFKC KVNNKDLPSP IERTISKIKG LVRAPQVYIL
241     PPPAEQLSRK DVSLTCLVVG FNPGDISVEW TSNGHTEENY KDTAPVLDSD GSYFIYSKLD
301     IKTSKWEKTD SFSCNVRHEG LKNYYLKKTI SRSPGK
```

IgG2c (A0A0A6YY53)

```
1       XKTTAPSVYP LAPVCGGTTG SSVTLGCLVK GYFPEPVTLT WNSGSLSSGV HTFPALLQSG
61      LYTLSSSVTV TSNTWPSQTI TCNVAHPASS TKVDKKIEPR VPITQNPCPP LKECPPCAAP
121     DLLGGPSVFI FPPKIKDVLM ISLSPMVTCV VVDVSEDDPD VQISWFVNNV EVHTAQTQTH
181     REDYNSTLRV VSALPIQHQD WMSGKEFKCK VNNRALPSPI EKTISKPRGP VRAPQVYVLP
241     PPAEEMTKKE FSLTCMITGF LPAEIAVDWT SNGRTEQNYK NTATVLDSDG SYFMYSKLRV
301     QKSTWERGSL FACSVVHEGL HNHLTTKTIS RSLGK
```

IgG3 (P03987)

```
1       TTTAPSVYPL VPGCSDTSGS SVTLGCLVKG YFPEPVTVKW NYGALSSGVR TVSSVLQSGF
61      YSLSSLVTVP SSTWPSQTVI CNVAHPASKT ELIKRIEPRI PKPSTPPGSS CPPGNILGGP
121     SVFIFPPKPK DALMISLTPK VTCVVVDVSE DDPDVHVSWF VDNKEVHTAW TQPREAQYNS
181     TFRVVSALPI QHQDWMRGKE FKCKVNNKAL PAPIERTISK PKGRAQTPQV YTIPPPREQM
241     SKKKVSLTCL VTNFFSEAIS VEWERNGELE QDYKNTPPIL DSDGTYFLYS KLTVDTDSWL
301     QGEIFTCSVV HEALHNHHTQ KNLSRSPGK
```

Figure S-2. Amino acid sequences of HC constant regions of murine IgG subclasses. Uniprot accession numbers of the secreted isoforms are given in brackets. The main cleavage products obtained upon digestion with SpeB are highlighted in grey; minor cleavage sites are marked with a blue line. *N*-glycosylation sites are highlighted in yellow; the C-terminal lysine residue (underlined) is usually absent in polyclonal IgGs but may be present in mAbs. Cysteine residues of intact intramolecular disulphide bonds identified after SpeB cleavage under reducing conditions are highlighted in light blue. Amino acid exchanges (N to D) identified in IgG1 (P01868) of BALB/c mice are highlighted in green (see fragment spectrum in Fig. S-3).

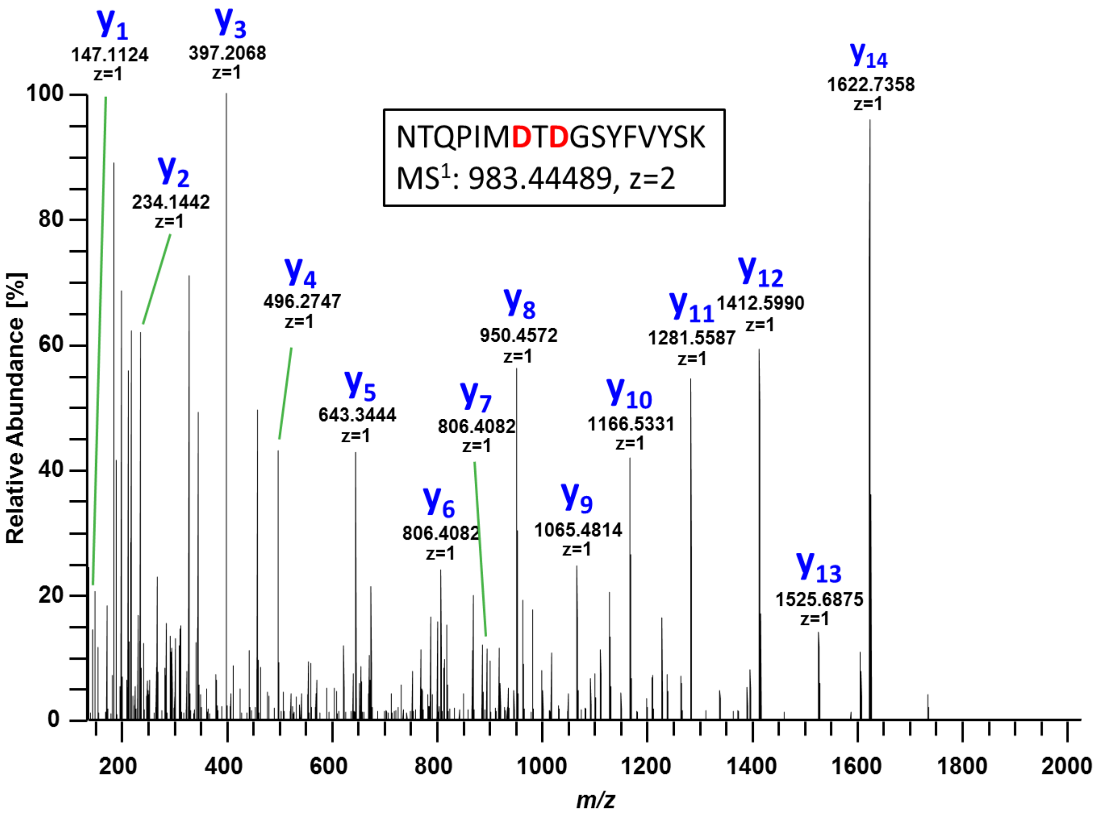

Figure S-3. Fragment ion spectrum of tryptic peptide [269-286] from the constant HC region of IgG1 (P01868, Fig. S-2) from BALB/c. Fragmentation was performed at 27.5 eV in the HCD cell; fragment ions were annotated with the help of GPMAW. Amino acid exchanges (red) are in accordance with a sequence conflict of P01868 listed in the Uniprot database.

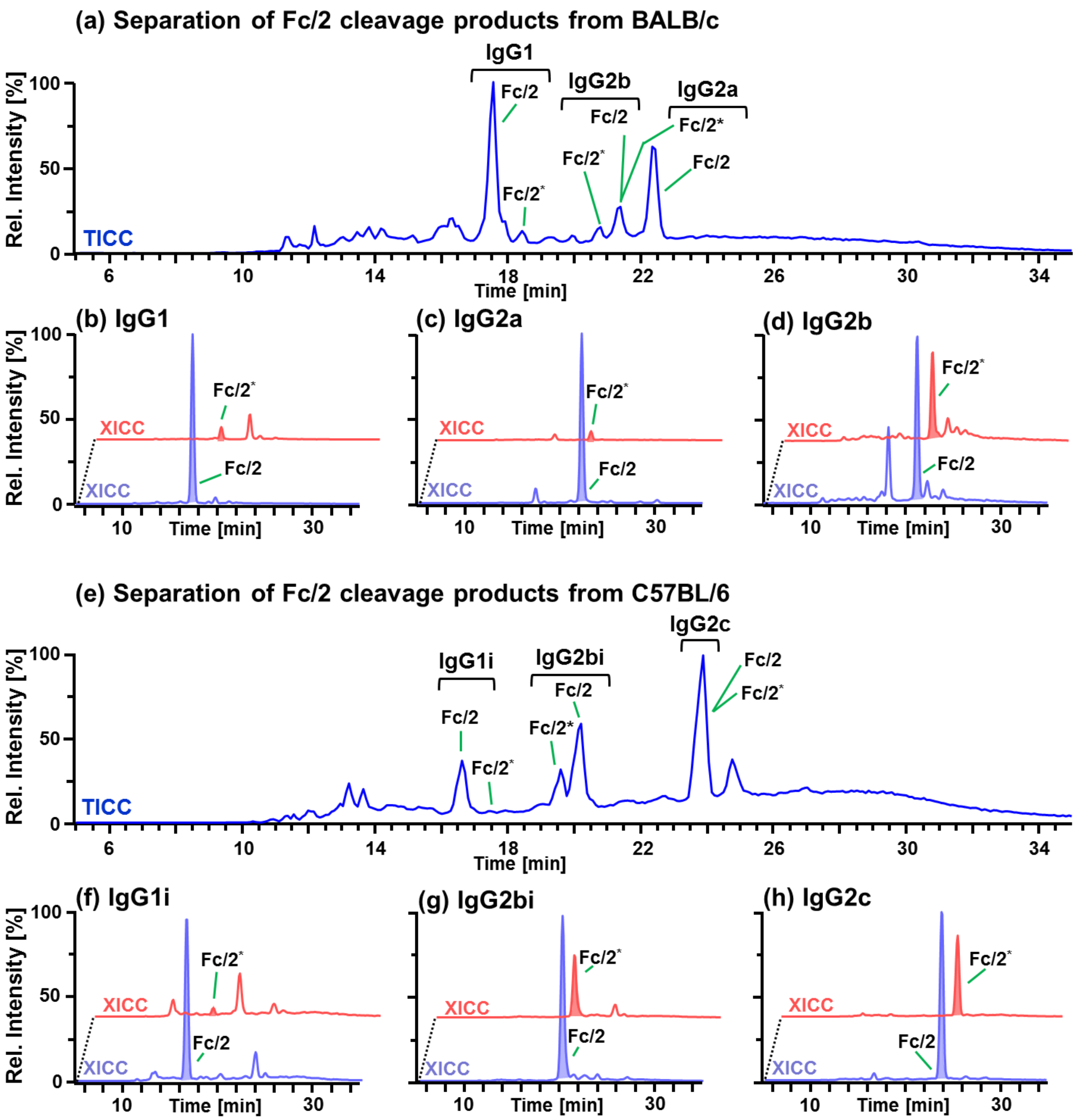

Figure S-4. Separation and quantification of Fc/2 cleavage products generated by SpeB. Main and minor cleavage products are designated Fc/2 and Fc/2*, respectively. (a) TICC of SpeB cleavage products of polyclonal IgGs from BALB/c. XICCs generated for Fc/2 and Fc/2* of (b) IgG1, (c) IgG2a and (d) IgG2b. (e) TICC of SpeB cleavage products of polyclonal IgGs from C57BL/6. XICCs generated for Fc/2 and Fc/2* of (f) IgG1i, (g) IgG2bi and (h) IgG2c. All XICCs were generated for G1F *N*-glycoforms lacking the C-terminal lysine residue; intramolecular disulphide bonds were considered to be intact. Peak areas used for relative quantification are indicated. Relative abundances of main and minor cleavage products are listed in Table S-2. XICCs for Fc/2* are depicted at a time offset of 2.0 min. Note that Fc/2 and Fc/2* cleavage products of each IgG subclass are chromatographically separated with the exception of IgG2c.

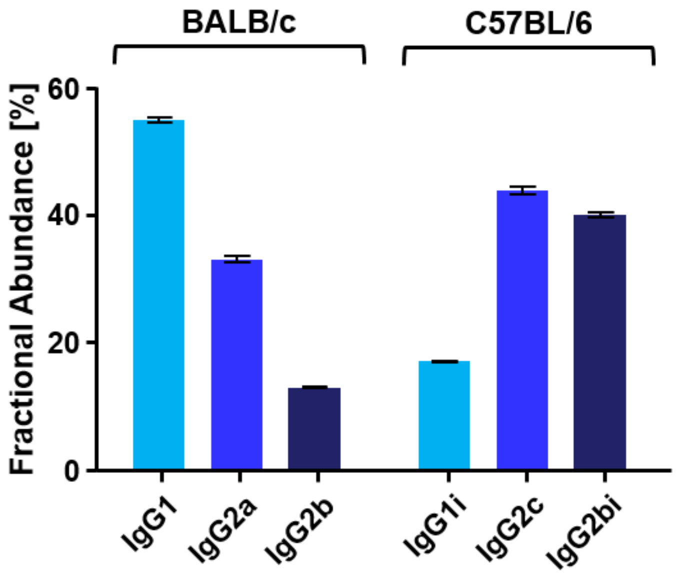

Figure S-5. Fractional subclass abundances of total IgGs from BALB/c and C57BL/6 mouse individuals. Relative abundances were obtained from XICCs of Fc/2 subunits including all detected glycoforms (Supplementary Excel file). Mean values are based on three technical replicates; error bars indicate standard deviations.

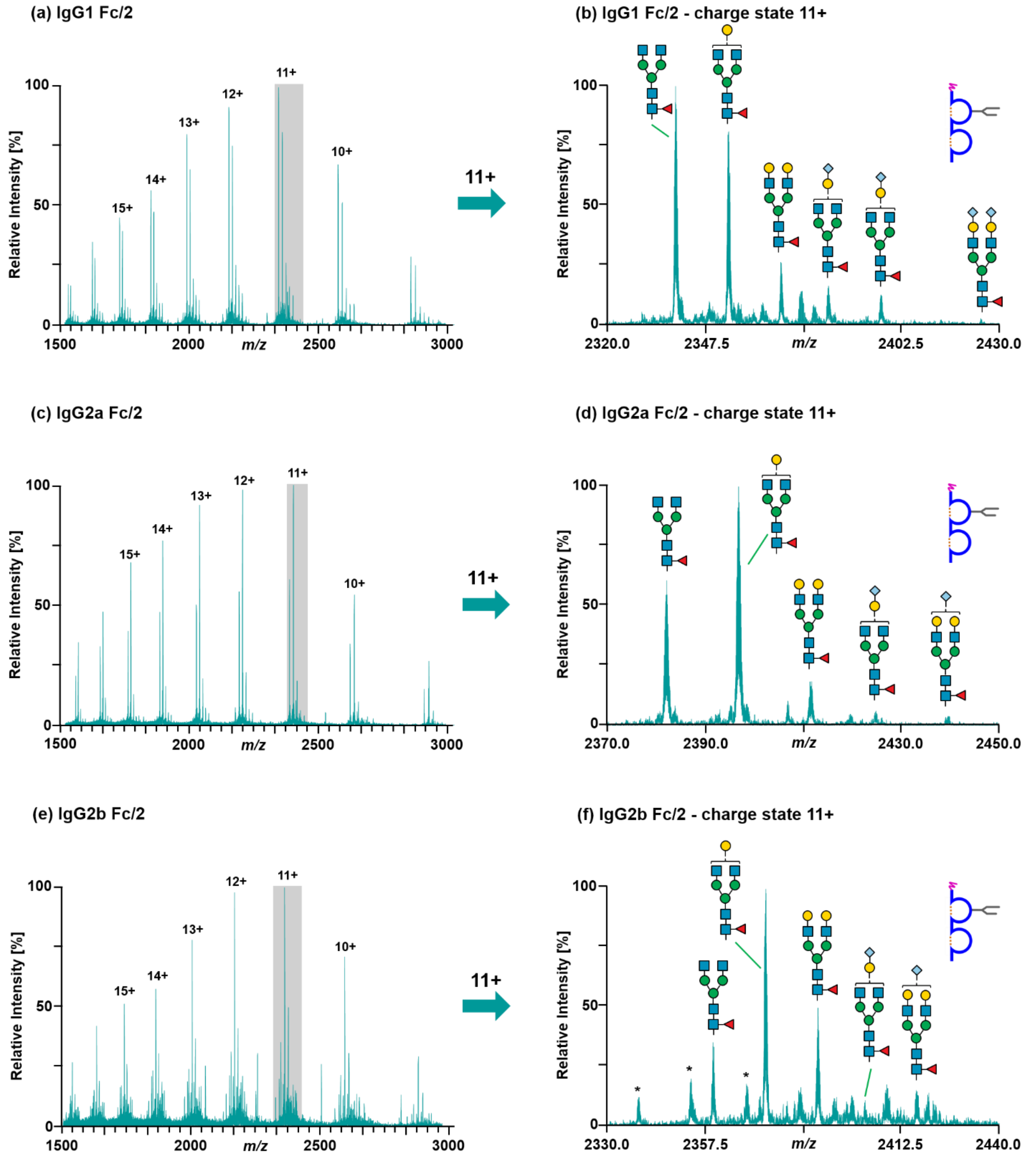

Figure S-6. Exemplary raw mass spectra of Fc/2 from (a) IgG1, (c) IgG2a and (e) IgG2b acquired in a total IgG preparation from a naive BALB/c mouse individual. Charge states used for generation of XICCs are indicated. Zooms of the most abundant charge states with assigned *N*-glycoforms are shown (b, d, f). Asterisks in (f) indicate masses assigned to co-eluting Fc/2* from IgG2a (Fig. S-4a). Note that the signal annotated with G2F may partially arise from the isobaric αG2F structure comprising α-gal.

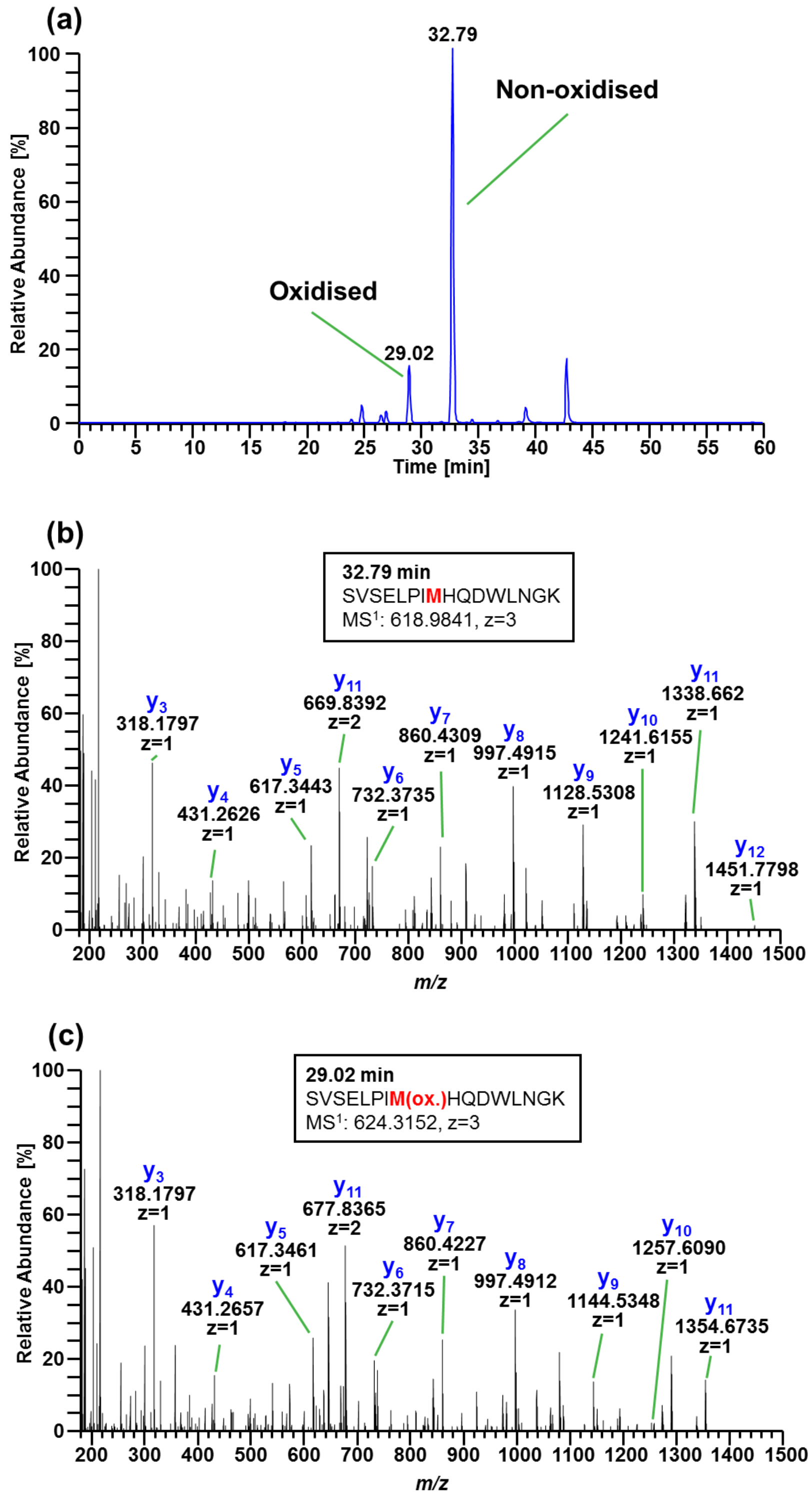

Figure S-7. Identification of oxidised tryptic peptide [179-194] from the constant HC region of IgG1 (P01868, Fig. S-2) from BALB/c. (a) XICC of non-oxidised and oxidised version of tryptic peptide [179-194]. The XICC was generated using the Xcalibur™ software taking into account doubly and triply charged ion species (non-oxidised: 618.6453 *m/z* and 927.4644 *m/z*; oxidised: 623.9770 *m/z* and 935.4618 *m/z;* isolation window ± 0.5 *m/z*). (b) Annotated fragment ion spectrum of the non-oxidised variant. (c) Annotated fragment ion spectrum of the oxidised variant. Fragmentation was performed at 27.5 eV in the HCD cell; fragment ions were annotated with the help of GPMAW.

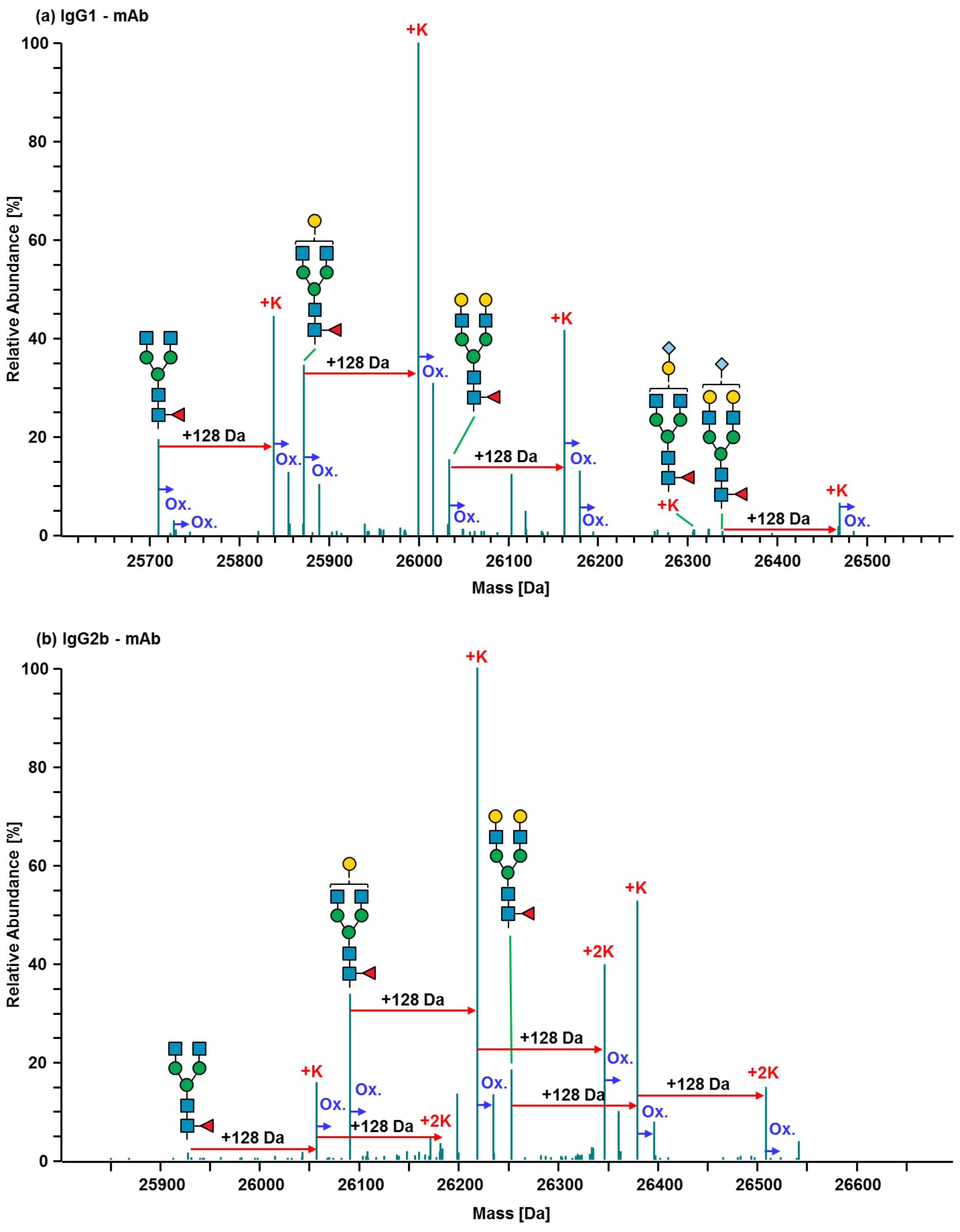

Figure S-8. Deconvoluted mass spectra of Fc/2 derived from (a) monoclonal murine IgG1 (P01868) and (b) monoclonal murine IgG2b (P01867). In addition to the main *N*-glycoforms, variants arising from oxidation (Ox.) or comprising additional lysine residues (+K, +2K) were identified based on characteristic mass shifts (+16 Da and +128 Da, respectively). Note that the signal annotated with G2F may partially arise from the isobaric αG2F structure comprising α-gal.

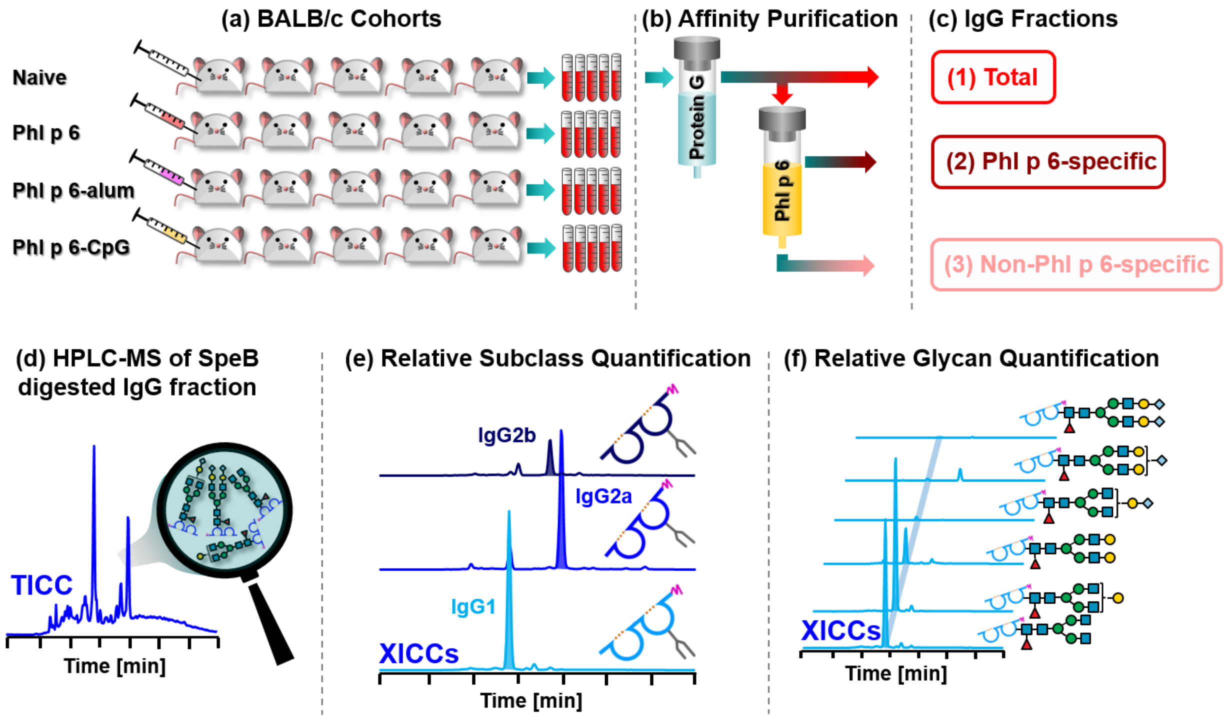

Figure S-9. Schematic workflow of the Phl p 6 vaccination study in BALB/c mice. (a) Serum was obtained from naive mice and mice treated with Phl p 6 in the absence or presence of adjuvants (alum or CpG), respectively (n=5). (b) Polyclonal IgGs of all subclasses were purified by protein G affinity chromatography; antigen-specific IgGs of vaccinated mice were affinity-purified from total IgGs using Phl p 6-sepharose. (c) Three IgG fractions were obtained for each vaccinated mouse individual. (d) SpeB digests of IgG fractions were analysed by means of HPLC-MS. XICCs of glycosylated Fc/2 cleavage products were generated to determine (e) relative subclass abundances and (f) relative *N*-glycoform abundances.

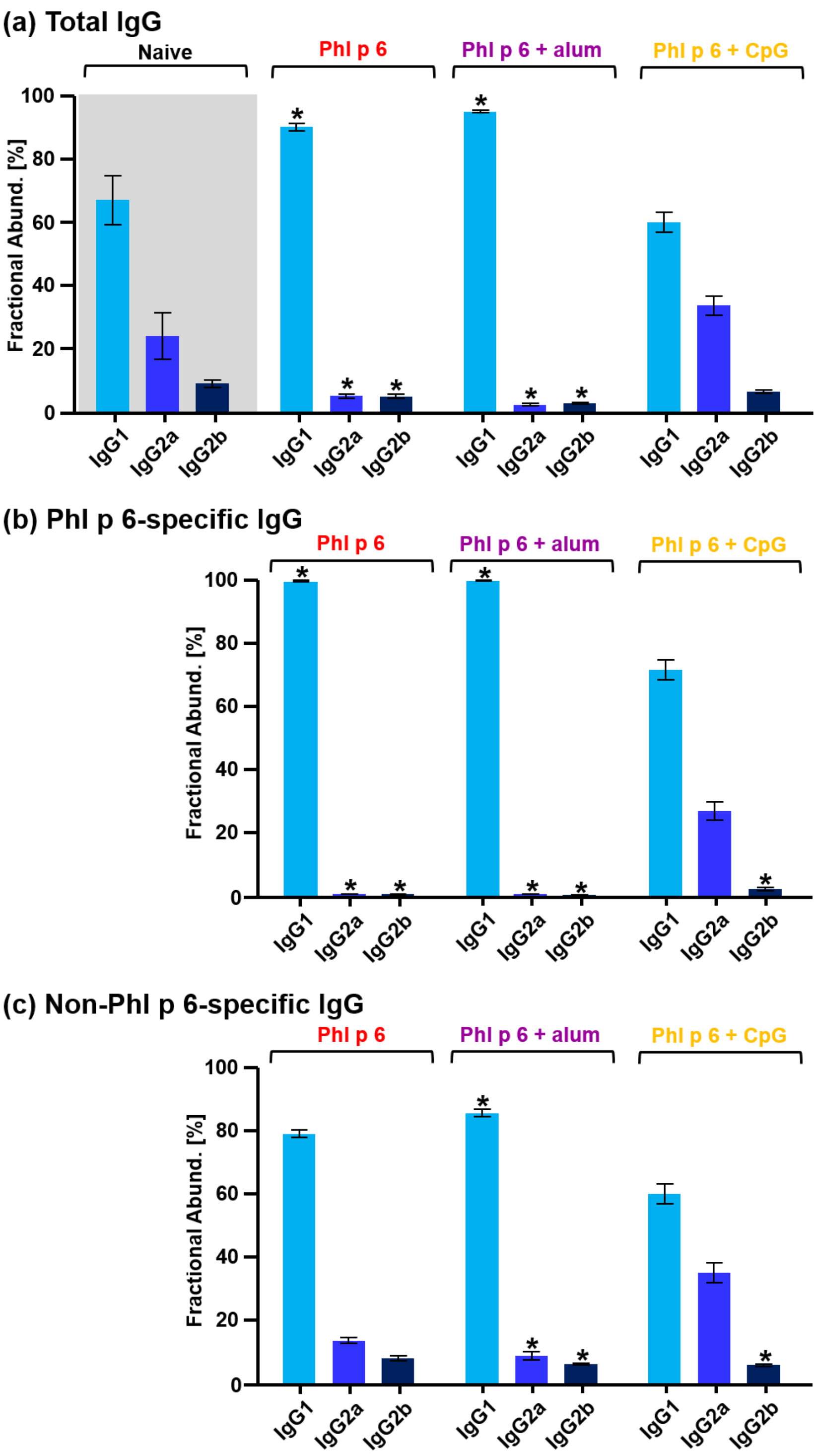

Figure S-10. Fractional abundances of IgG subclasses in (a) total IgG, (b) antigen-specific IgG and (c) antigen-specific depleted IgG from BALB/c mice. Abundances are shown for naive mice and cohorts vaccinated with Phl p 6, Phl p 6 + alum and Phl p 6 + CpG. Relative quantification was based on XICCs of Fc/2 subunits including all detected glycoforms. Mean values of five mouse individuals including standard deviations are shown (n=5) (Supplementary Excel file). Statistical significances were tested using a one-way ANOVA with a Dunnett post-hoc-test using naive mice as a control. Significant changes are indicated with an asterisk ($p \leq 0.05$).

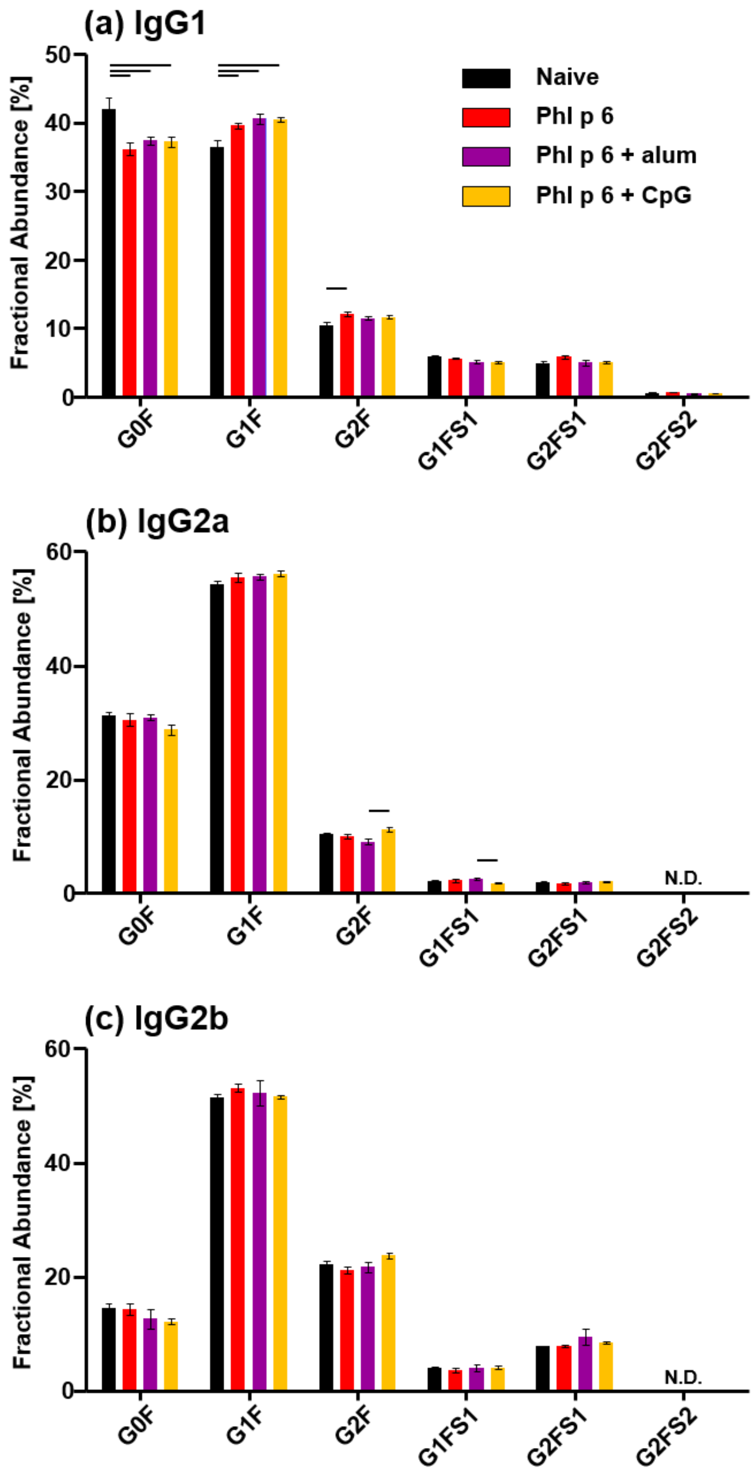

Figure S-11. Subclass-specific glycosylation profiles of total IgGs from naive and Phl p 6-vaccinated BALB/c mice. Relative glycoform abundances of (a) IgG1, (b) IgG2a and (c) IgG2b in naive mice and cohorts treated with Phl p 6, Phl p 6 + alum and Phl p 6 + CpG, respectively, are shown. Fractional abundances represent mean values of five mouse individuals including standard deviations (n=5) (Supplementary Excel file). Statistical significances were tested using a one-way ANOVA with a Tukey post-hoc-test. Significant changes are indicated ($p \leq 0.05$). Note that the abundances of G2F may partially arise from the isobaric αG2F structure comprising α-gal.

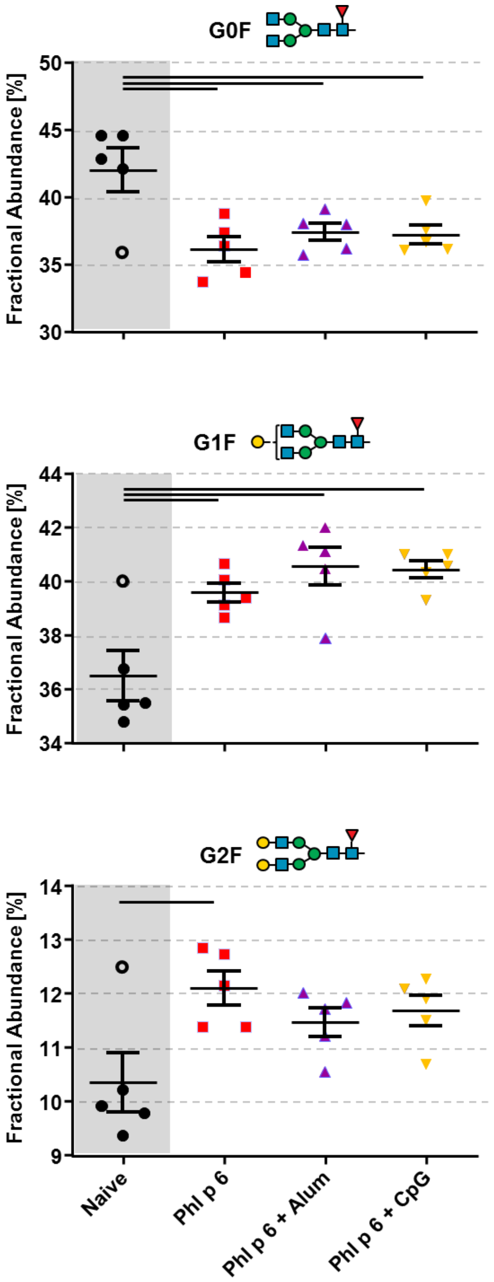

Figure S-12. G0F, G1F and G2F glycoform abundances of IgG1 in total IgGs from naive and Phl p 6-vaccinated BALB/c mouse individuals. Data corresponds to the bar chart shown in Figure S-10 (a). Mean values of five mouse individuals including standard deviations are shown. The outlier individual in the naive cohort is indicated as open circle. Statistical significances were tested using a one-way ANOVA with a Tukey post-hoc-test. Significant changes are indicated ($p \leq 0.05$). Note that the abundances of G2F may partially arise from the isobaric αG2F structure comprising α-gal.

Table S-1. SpeB cleavage products of murine IgG heavy chains from different sources. Main and minor cleavage products are designated Fc/2 and Fc/2*, respectively. Theoretical monoisotopic masses were calculated for G1F *N*-glycoforms lacking the C-terminal lysine residue; intramolecular disulphide bonds were considered to be intact. Experimental masses are mean values of three technical replicates.
[a]Theoretical monoisotopic masses were calculated using GPMAW. [b]Experimental monoisotopic masses were obtained by deconvolution using the Xtract algorithm implemented in the Xcalibur™ software. [c]Standard deviations were calculated from three technical replicates. [d]The low mass accuracy may be attributed to a previously described +1 Da shift arising from an isotopic misassignment during deconvolution.[3]

	Sub-class	Uniprot ID	Product	Amino acids	Fractional abundance [%]	Theoretical mass [Da][a]	Experimental mass [Da][b]	Standard mass deviation [mDa][c]	Mass accuracy [ppm]
BALB/c	IgG1	P01868	Fc/2	110-323	93.04	25,871.57	25,871.50	5.03	-2.58
			Fc/2*	108-323	6.96	26,087.66	26087.87	15.37	3.96
	IgG2a	P01863	Fc/2	112-329	95.30	26,339.87	26,340.85	2.00	37.20[d]
			Fc/2*	117-239	4.70	25,857.68	25,858.71	3.61	39.83[d]
	IgG2b	P01867	Fc/2	117-334	64.28	26,088.91	26,089.87	1.53	36.92[d]
			Fc/2*	116-334	35.72	26,217.00	26,217.01	4.04	0.25
C57BL/6	IgG1i	A0A075B5P4	Fc/2	110-323	96.44	25,836.64	25,836.60	3.61	-1.55
			Fc/2*	108-323	3.56	26,052.73	26,052.74	14.73	0.38
	IgG2bi	A0A075B5P3	Fc/2	118-335	71.69	26,069.97	26,070.98	1.15	38.87[d]
			Fc/2*	117-335	28.31	26,198.06	26,198.05	1.00	-0.38
	IgG2c	A0A0A6YY53	Fc/2	118-334	68.07	25,794.80	25,794.81	5.51	0.52
			Fc/2*	119-334	31.93	25,723.76	25,723.76	0.58	0.00
mAb	IgG3	P03987	Fc/2*	109-329	70.90	26,394.03	26,394.00	8.08	-1.26
			Fc/2	105-329	29.10	26,746.21	26,746.19	1.53	-0.87

Table S-2. *N*-glycan structures considered in glycoform assignment using MoFi software.[4] The library is based on glycopeptide data for murine IgGs reported in recent literature.[5]

Structure	Abbreviation	Elemental composition	Monosaccharide composition	Monoisotopic mass [Da]
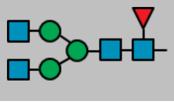	G0F	H92C56N4O39	HexNAc$_4$ Hex$_3$ Fuc	1444.53387
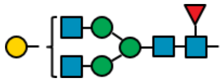	G1F	H102C62N4O44	HexNAc$_4$ Hex$_4$ Fuc	1606.58669
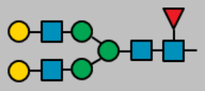	G2F	H112C68N4O49	HexNAc$_4$ Hex$_5$ Fuc	1768.63952
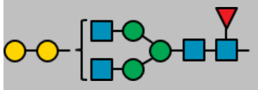	αG2F			
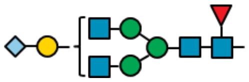	G1FS1	H119C73N5O53	HexNAc$_4$ Hex$_4$ Fuc Neu5Gc	1913.67702
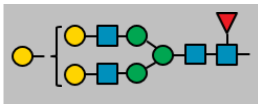	αG3F	H122C74N4O54	HexNAc$_4$ Hex$_6$ Fuc	1930.69236
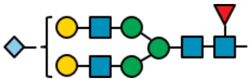	G2FS1	H129C79N5O58	HexNAc$_4$ Hex$_5$ Fuc Neu5Gc	2075.72985
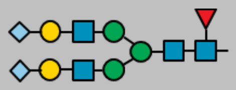	G2FS2	H148C90N6O67	HexNAc$_4$ Hex$_5$ Fuc Neu5Gc$_2$	2384.83583
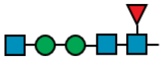		H66C40N2O29	HexNAc$_3$ Hex$_2$ Fuc	1038.37512
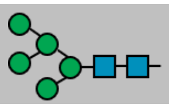		H76C46N2O35	HexNAc$_2$ Hex$_5$	1216.42286
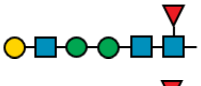 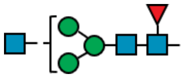		H79C48N3O34	HexNAc$_3$ Hex$_3$ Fuc	1241.45450
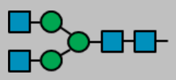	G0	H82C50N4O35	HexNAc$_4$ Hex$_3$	1298.47596

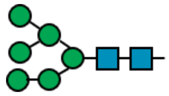		H86C52N2O40	HexNAc$_2$ Hex$_6$	1378.47569
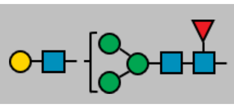		H89C54N3O39	HexNAc$_3$ Hex$_4$ Fuc	1403.50732
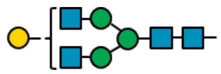	G1	H92C56N4O40	HexNAc4 Hex4	1460.52878
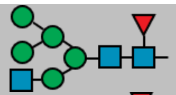 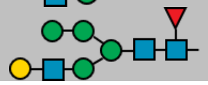		H99C60N3O44	HexNAc$_3$ Hex$_5$ Fuc	1565.56014
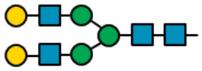	G2	H102C62N4O45	HexNAc$_4$ Hex$_5$	1622.58161
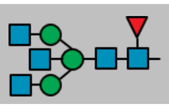		H105C64N5O44	HexNAc$_5$ Hex$_3$ Fuc	1647.61325
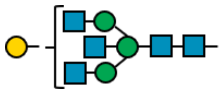		H105C64N5O45	HexNAc$_5$ Hex$_4$	1663.60816
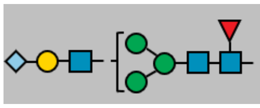		H106C65N4O48	HexNAc$_3$ Hex$_4$ Fuc Neu5Gc	1710.59765
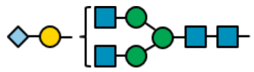		H109C67N5O49	HexNAc$_4$ Hex$_4$ Neu5Gc	1767.61911
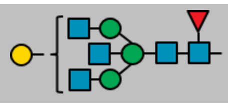		H115C70N5O49	HexNAc$_5$ Hex$_6$ Fuc	1809.66608
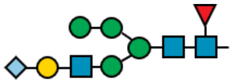		H116C71N4O53	HexNAc$_3$ Hex$_5$ Fuc Neu5Gc	1872.65047
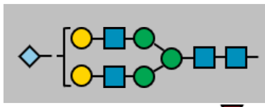		H119C73N5O54	HexNAc$_4$ Hex$_5$ Neu5Gc	1929.67194
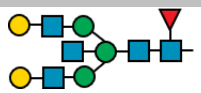		H125C76N5O54	HexNAc$_5$ Hex$_5$ Fuc	1971.71889
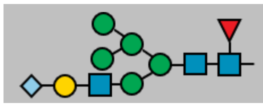		H126C77N4O58	HexNAc$_3$ Hex$_6$ Fuc Neu5Gc$_1$	2034.70332
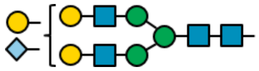		H129C79N5O59	HexNAc$_4$ Hex$_6$ Neu5Gc$_1$	2091.72476
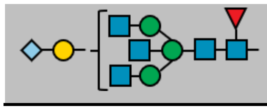		H132C81N6O58	HexNAc$_5$ Hex$_4$ Fuc Neu5Gc	2116.75640

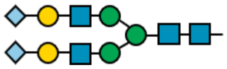	H136C84N6O63	$HexNAc_4$ Hex_5 $Neu5Gc_2$	2236.76227
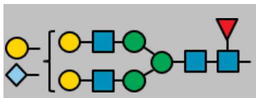	H139C85N5O63	$HexNAc_4$ Hex_6 Fuc $Neu5Gc_1$	2237.78269
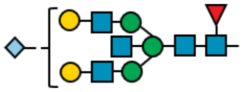	H142C87N6O63	$HexNAc_5$ Hex_5 Fuc Neu5Gc	2278.80922
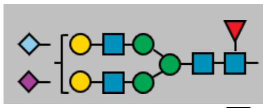	H146C90N6O66	$HexNAc_4$ Hex_5 Fuc Neu5Gc Neu5Ac	2366.82526
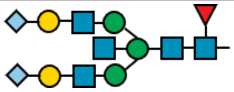	H159C98N7O72	$HexNAc_5$ Hex_5 Fuc $Neu5Gc_2$	2585.89955

SI References

1 Fellers, R. T. *et al.* ProSight Lite: graphical software to analyze top-down mass spectrometry data. *Proteomics* **15**, 1235-1238 (2015).

2 Peri, S., Steen, H. & Pandey, A. GPMAW--a software tool for analyzing proteins and peptides. *Trends Biochem Sci* **26**, 687-689 (2001).

3 Senko, M. W., Beu, S. C. & McLafferty, F. W. Determination of monoisotopic masses and ion populations for large biomolecules from resolved isotopic distributions. *Journal of the American Society for Mass Spectrometry* **6**, 229-233 (1995).

4 Skala, W., Wohlschlager, T., Senn, S., Huber, G. E. & Huber, C. G. MoFi: A Software Tool for Annotating Glycoprotein Mass Spectra by Integrating Hybrid Data from the Intact Protein and Glycopeptide Level. *Anal Chem* **90**, 5728-5736, doi:10.1021/acs.analchem.8b00019 (2018).

5 de Haan, N. *et al.* The N-Glycosylation of Mouse Immunoglobulin G (IgG)-Fragment Crystallizable Differs Between IgG Subclasses and Strains. *Front Immunol* **8**, 608, doi:10.3389/fimmu.2017.00608 (2017).
